# Applying ChatGPT in public health: a SWOT and PESTLE analysis

**DOI:** 10.3389/fpubh.2023.1225861

**Published:** 2023-07-03

**Authors:** Plinio P. Morita, Shahabeddin Abhari, Jasleen Kaur, Matheus Lotto, Pedro Augusto Da Silva E. Souza Miranda, Arlene Oetomo

**Affiliations:** ^1^School of Public Health Sciences, University of Waterloo, Waterloo, ON, Canada; ^2^Department of Systems Design Engineering, University of Waterloo, Waterloo, ON, Canada; ^3^Research Institute for Aging, University of Waterloo, Waterloo, ON, Canada; ^4^Centre for Digital Therapeutics, Techna Institute, University Health Network, Toronto, ON, Canada; ^5^Dalla Lana School of Public Health, Institute of Health Policy, Management, and Evaluation, University of Toronto, Toronto, ON, Canada; ^6^Department of Pediatric Dentistry, Orthodontics, and Public Health, Bauru School of Dentistry, University of São Paulo, Bauru, Brazil

**Keywords:** public health, artificial intelligence, ChatGPT, SWOT analysis, healthcare

## 1. Introduction

Public health is a multidisciplinary field that aims to promote and protect the health of communities through various interventions, such as disease prevention, health promotion, and policy development. It involves analyzing data and applying evidence-based approaches to improve the health outcomes of populations (1–3). One of the main challenges in public health is the emergence and re-emergence of infectious diseases such as COVID-19, which can pose a significant threat to public health (4). Other challenges include the rise of non-communicable diseases (NCDs) such as heart disease and cancer, which are often linked to lifestyle factors such as poor diet and physical inactivity (5). Health inequities also pose a challenge to public health, as some groups, such as marginalized and vulnerable populations, may experience poorer health outcomes due to commercial determinants of health (1, 3, 5). Furthermore, there are challenges associated with the collection, management, and analysis of public health data. Ensuring data privacy and security, addressing biases in data collection and analysis, and making data accessible to all stakeholders are all critical issues in the field of public health. Finally, the need for effective communication and collaboration among stakeholders is essential to address these challenges and improve public health outcomes (2, 6, 7).

The growth of Artificial Intelligence (AI) in healthcare has been exponential in recent years, with advancements in machine learning, natural language processing (NLP), and image analysis. AI is increasingly being used to improve disease surveillance, drug discovery, and personalized medicine, among other applications, with the potential to transform healthcare delivery (8–10). Also, AI methods have shown great promise in addressing various public health challenges. Machine learning algorithms, NLP, and other AI techniques can be used to analyze large datasets, identify patterns and trends, and generate insights that can inform public health interventions (11, 12). ChatGPT is a state-of-the-art NLP model developed by OpenAI that has shown impressive performance in a variety of tasks, including language translation, text completion, and sentiment analysis. The model's ability to generate coherent and contextually appropriate responses to text inputs has made it a promising tool for a wide range of applications, including public health (13). For example, ChatGPT can be used to help patients manage chronic conditions by providing reminders for medication, diet, and exercise, and answering questions about symptoms and treatment options. ChatGPT can also be used to help patients find healthcare providers, schedule appointments, and access healthcare information. Moreover, ChatGPT can be used to improve patient engagement and education. Patients can interact with ChatGPT in natural language and receive tailored responses based on their medical history, preferences, and needs. ChatGPT can also provide patients with reliable and up-to-date health information, such as disease prevention tips, symptom management advice, and resources for mental health support (13–15).

In this paper, we conducted a SWOT analysis and PESTLE analysis (16, 17) to evaluate the applying of ChatGPT in public health. SWOT stands for Strengths, Weaknesses, Opportunities, and Threats, and is a widely used strategic planning tool that helps in identifying the internal and external factors that can impact the success of a project or initiative (17). As well as PESTLE analysis is a strategic planning tool used to assess and analyze external factors that can impact an organization, project, or industry. It examines the Political, Economic, Sociocultural, Technological, Legal, and Environmental factors that can influence the environment in which an entity operates (16). By conducting a SWOT analysis of ChatGPT, we aim to identify the strengths, weaknesses, opportunities, and threats associated with the application of this technology in public health. Also, we want to determine main external factors that can have an effect on applying this technology in public health.

The methods used in this paper involve a comprehensive literature review of previous studies and publications related to the applications of ChatGPT in public health (18). To identify pertinent studies for our research, a comprehensive search was conducted in reputable databases, employing relevant keywords. The databases used for this purpose were Pubmed, Scopus, and Google Scholar. The selection of relevant articles was based on a combination of crucial keywords that proved effective in narrowing down the search results. The selected keywords for this study were “ChatGPT” AND (“public health” OR “healthcare”). To ensure consistency and focus, the search was limited to English-language papers published before April 15, 2023. Through this systematic approach, a total of 106 articles were initially identified across the three aforementioned databases. After evaluation of these articles based on their alignment with the research topic, 16 papers were ultimately deemed relevant and included for further analysis. After identifying relevant articles, a qualitative content analysis was conducted to extract and classify relevant information related to the SWOT analysis and PESTLE analysis of ChatGPT in public health.

## 2. SWOT analysis for applying ChatGPT in public health

The extracted data were organized into four categories: strengths, weaknesses, opportunities, and threats.

### 2.1. Strengths

The use of ChatGPT in public health has several strengths. One of the main strengths of ChatGPT is its ability to provide personalized health information and support to individuals. Chatbots powered by ChatGPT can be available 24/7, which can improve access to health information and support for people who may not be able to seek care during regular business hours. Additionally, ChatGPT can process and analyze large amounts of data quickly and accurately, which can support disease surveillance and outbreak detection. Chatbots can monitor social media and other online platforms for signs of emerging health threats, such as outbreaks of infectious diseases. They can also provide real-time information to individuals and healthcare providers about outbreaks in their area, which can help to prevent the spread of disease (13–15, 19–23).

### 2.2. Weaknesses

The use of ChatGPT in public health also has several weaknesses. One of the main weaknesses is the potential for misinterpretation or miscommunication, as language models may not always accurately understand the nuances of human language and context. This could result in chatbots providing incorrect or misleading health information (22, 24). Additionally, privacy is a concern, as chatbots may be vulnerable to hacking or data breaches, which can compromise sensitive health information. There is also the potential for ChatGPT to perpetuate biases in health data if the underlying data used to train the model is biased. For example, if the data used to train ChatGPT is biased toward certain demographics, the chatbot may not provide accurate information to all populations (15, 20–28).

### 2.3. Opportunities

There are several opportunities associated with the use of ChatGPT in public health. One of the main opportunities is the ability of chatbots to provide personalized health information and support to individuals. This is particularly useful for individuals who may not have access to healthcare services or who may be hesitant to seek care due to stigma or other barriers (11, 13, 21, 27). ChatGPT can also assist with disease surveillance and epidemic identification, which helps stop the spread of disease. Chatbots can keep an eye on social media and other online platforms for indications of emerging health risks, such infectious disease epidemics. They can also notify people and healthcare professionals in real time about epidemics in their region. Another opportunity is that ChatGPT can facilitate communication and collaboration between healthcare providers and patients, which can improve the quality of care and health outcomes (11, 13, 15, 21–25).

### 2.4. Threats

**Figure 1:**
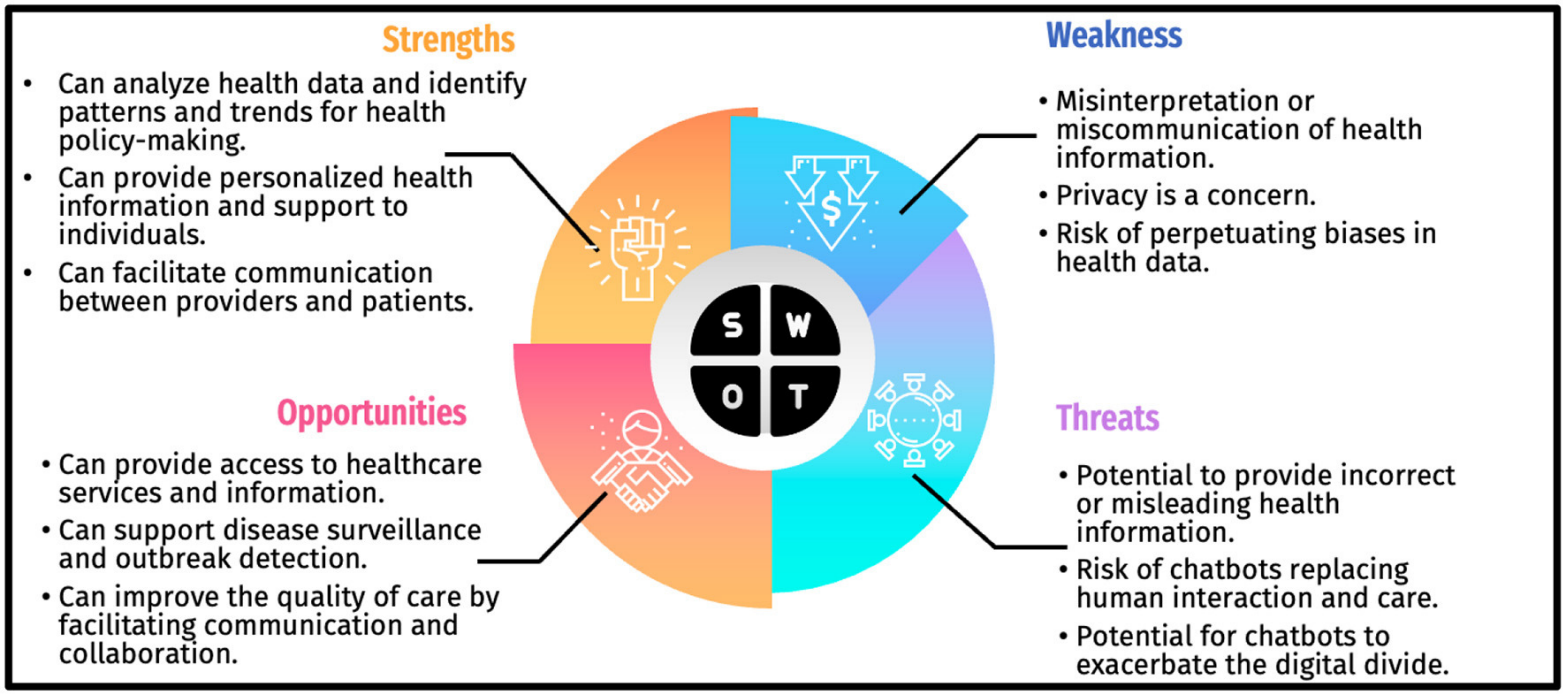
SWOT analysis for applying chatbot in public health.

The use of ChatGPT in public health also has several threats. The possibility of chatbots distributing inaccurate or deceptive health information is one of the key dangers. This can be as a result of biased training data or mistakes in the language model's comprehension of human language and context (11, 14, 21, 24). Additionally, as was already said, there is a chance that chatbots would maintain the biases that now exist in health data. Another danger is that chatbots may take the role of human connection and care, which would reduce empathy and prevent personalized care from being provided. Finally, there is a chance that chatbots will widen the digital divide by excluding those without access to technology or who are uncomfortable utilizing it from ChatGPT's public health advantages (9–11, 21–24, 26–29). The summary of SWOT analysis showed in Figure 1.

## 3. PESTLE analysis for applying ChatGPT in public health

The application of ChatGPT in public health can be analyzed using the PESTLE framework, which examines the Political, Economic, Sociocultural, Technological, Legal, and Environmental factors that influence its implementation and impact. This analysis aims to provide a comprehensive understanding of the external factors that shape the context of ChatGPT's application in public health.

(1) The Political factor explores the political environment surrounding the use of ChatGPT in public health. This includes government policies, regulations, and political support or resistance to AI technologies. Political factors play a crucial role in determining the level of investment, data governance, and ethical considerations in deploying ChatGPT in public health (24, 28, 30).
(2) The Economic factor examines the economic implications of using ChatGPT in public health. This involves evaluating the cost-effectiveness, affordability, and sustainability of implementing the technology. Economic factors also consider the potential for job displacement or creation, economic disparities in access to AI technologies, and the overall financial implications for healthcare systems (23, 24, 27, 29, 30).
(3) Sociocultural factors play a significant role in the application of ChatGPT in public health. These factors involve understanding public acceptance, trust, and perception of AI technologies. Sociocultural considerations also encompass issues of privacy, data security, and the potential impact on the patient-provider relationship. Moreover, cultural norms, beliefs, and attitudes toward AI-driven healthcare interventions need to be taken into account (15, 23, 27, 30).
(4) The Technological factor analyzes the technological landscape for ChatGPT in public health. This includes advancements in natural language processing, machine learning algorithms, and the integration of ChatGPT with existing health information systems. Technological factors also encompass the potential for bias, algorithmic transparency, and the need for continuous updates and maintenance of the technology (22, 27, 29, 30).
(5) Legal factors examine the legal and regulatory framework governing the use of ChatGPT in public health. This includes privacy regulations, data protection laws, intellectual property rights, and ethical guidelines for AI applications in healthcare. Compliance with legal requirements and adherence to ethical principles are critical for the responsible deployment of ChatGPT in public health (14, 27, 30).
(6) The Environmental factor focuses on the environmental implications of implementing ChatGPT in public health. This involves considering the energy consumption and carbon footprint associated with AI infrastructure and data centers. It also includes assessing the environmental impact of data collection, storage, and disposal practices (23, 31, 32).

## 4. Discussion

The findings highlight the potential benefits and limitations of using chatbots powered by ChatGPT in the public health context. Consequently, they can provide personalized health information and support to individuals, disease surveillance, and outbreak detection, besides facilitating individual and shared decision-making. Nevertheless, there are also limitations associated with ChatGPT that should be considered, such as the potential misinterpretation or miscommunication, privacy concerns, and the risk of perpetuating biases in health data. By conducting a PESTLE analysis, policymakers, healthcare organizations, and researchers can gain insights into the broader contextual factors that can influence the application of ChatGPT in public health. This analysis can inform decision-making, help anticipate challenges, and guide the development of ethical guidelines and regulatory frameworks to maximize the benefits and mitigate potential risks associated with ChatGPT's implementation in the public health domain.

Indeed, future studies must clarify whether the advantages of utilizing chatbots outweigh their risks in assisting public health actions. Although artificial intelligence-based approaches can improve healthcare outcomes, the threats should be carefully considered to avoid inappropriate decision-making and the deepening of health inequalities especially the digital divide which continues to grow especially in the Global South. Hence, the widespread adoption of the disruptive technology of AI chatbots in public health will require careful oversight and time as authorities must first understand the optimal scenarios for the ethics and legalities of its implementation and application. However, these actions cannot be delayed because hundreds of AI tools are being released and are being used for learning about public health whether by design or not.

## Author contributions

PPM and SA: conceptualization and writing original draft. JK, SA, ML, PM, and AO: writing review and editing. PPM, SA, JK, ML, PM, and AO: conceptualization, supervision, and writing review and editing. All authors contributed to the article and approved the submitted version.
